# Supplementary figures and images for: Energy-efficient routing protocol for reliable low-latency Internet of Things in oil and gas pipeline monitoring
Source: PeerJ Comput Sci. 2024 Feb 29;10:e1908. doi: 10.7717/peerj-cs.1908 (PMC10909229; doi:10.7717/peerj-cs.1908)

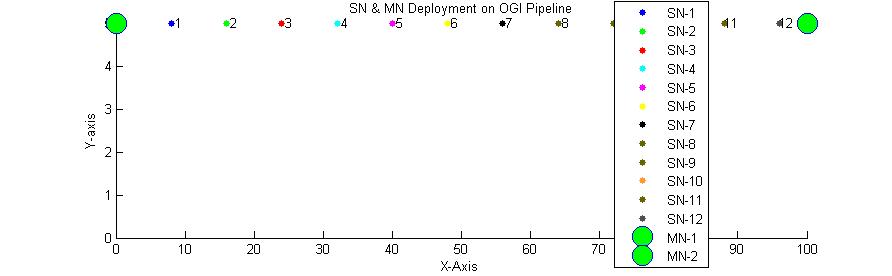

Supplement: Supplemental Information 1 [file peerj-cs-10-1908-s001.zip › IoT-OGI-code/OGI-code/Depl.jpg]

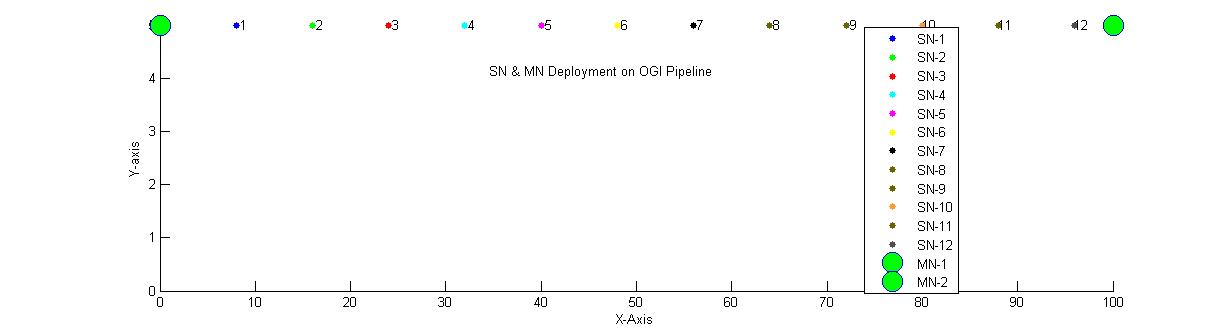

Supplement: Supplemental Information 1 [file peerj-cs-10-1908-s001.zip › IoT-OGI-code/OGI-code/figure/Depl.jpg]

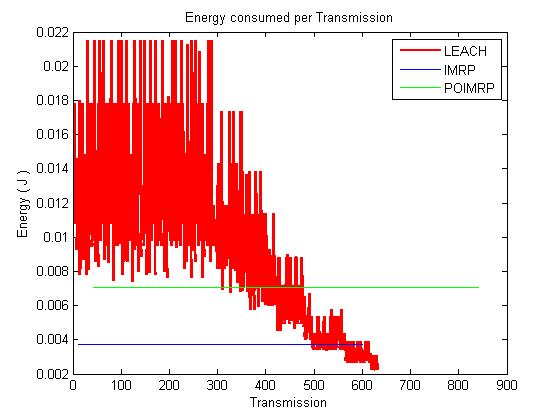

Supplement: Supplemental Information 1 [file peerj-cs-10-1908-s001.zip › IoT-OGI-code/OGI-code/figure/energy consumed vs transmissions-all 3 comp(concise).jpg]

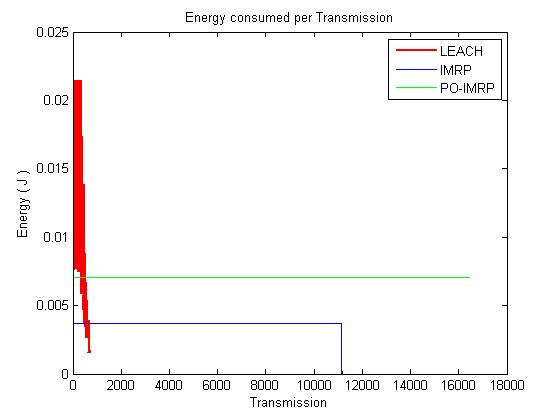

Supplement: Supplemental Information 1 [file peerj-cs-10-1908-s001.zip › IoT-OGI-code/OGI-code/figure/energy consumed vs transmissions-leach comp.jpg]

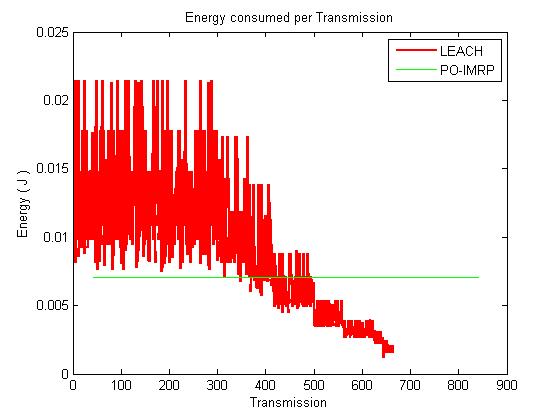

Supplement: Supplemental Information 1 [file peerj-cs-10-1908-s001.zip › IoT-OGI-code/OGI-code/figure/energy consumed vs transmissions-leach vs POIMRP (concise).jpg]

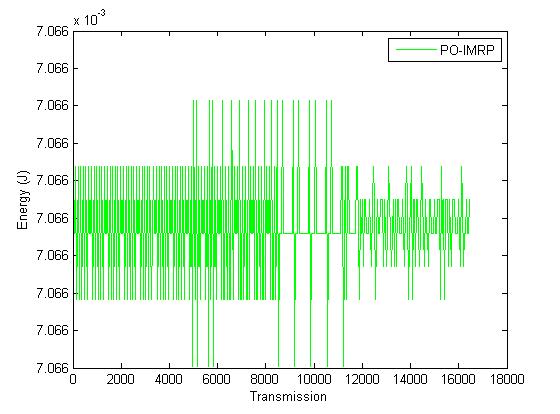

Supplement: Supplemental Information 1 [file peerj-cs-10-1908-s001.zip › IoT-OGI-code/OGI-code/figure/Energy consumed vs transmissions-POIMRP.jpg]

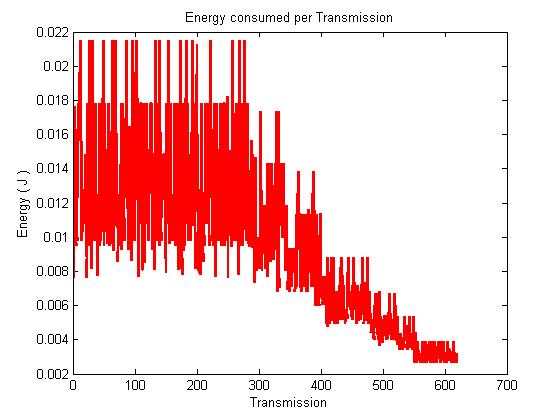

Supplement: Supplemental Information 1 [file peerj-cs-10-1908-s001.zip › IoT-OGI-code/OGI-code/figure/energy consumed vs trx-leach only.jpg]

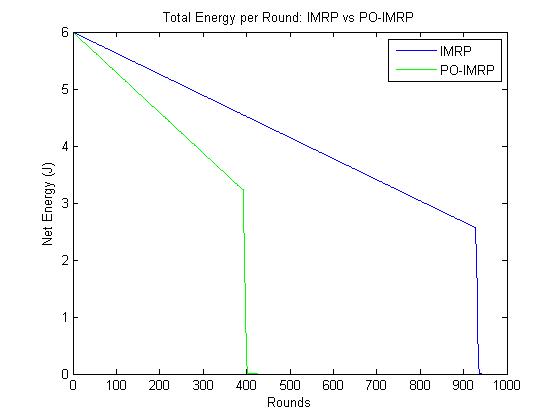

Supplement: Supplemental Information 1 [file peerj-cs-10-1908-s001.zip › IoT-OGI-code/OGI-code/figure/Net Energyper round.jpg]

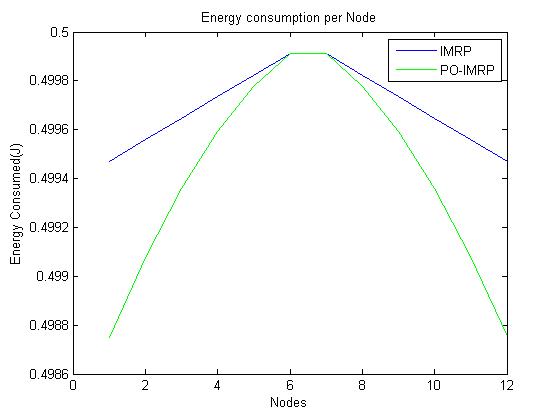

Supplement: Supplemental Information 1 [file peerj-cs-10-1908-s001.zip › IoT-OGI-code/OGI-code/figure/nodes vs energy at cold start for a single round.jpg]

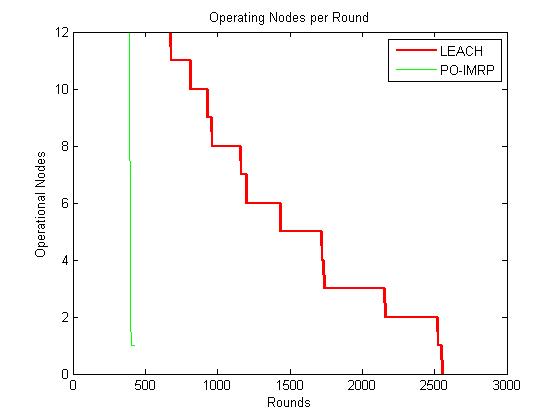

Supplement: Supplemental Information 1 [file peerj-cs-10-1908-s001.zip › IoT-OGI-code/OGI-code/figure/op nodes vs rounds.jpg]

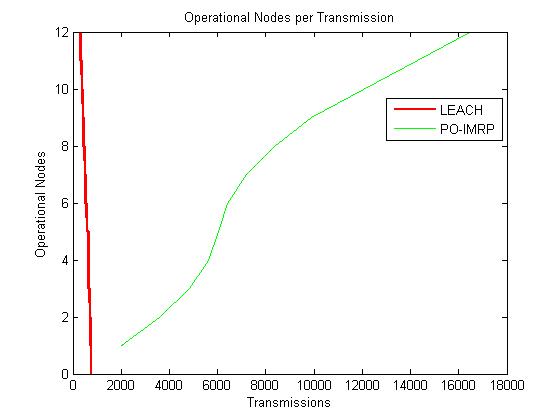

Supplement: Supplemental Information 1 [file peerj-cs-10-1908-s001.zip › IoT-OGI-code/OGI-code/figure/op nodes vs transmissions -leach comp.jpg]

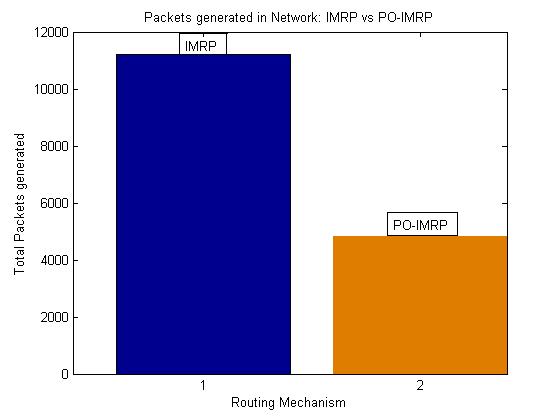

Supplement: Supplemental Information 1 [file peerj-cs-10-1908-s001.zip › IoT-OGI-code/OGI-code/figure/packet-generated vs mech.jpg]

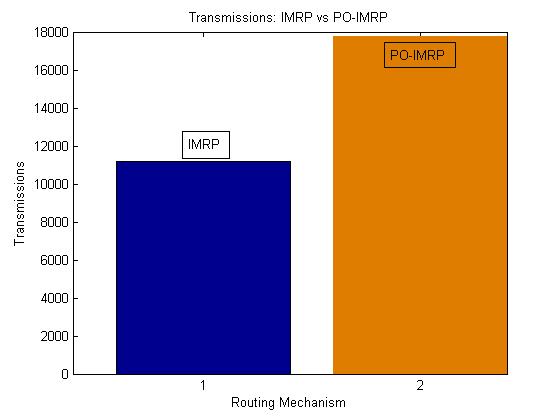

Supplement: Supplemental Information 1 [file peerj-cs-10-1908-s001.zip › IoT-OGI-code/OGI-code/figure/packets vs mech.jpg]

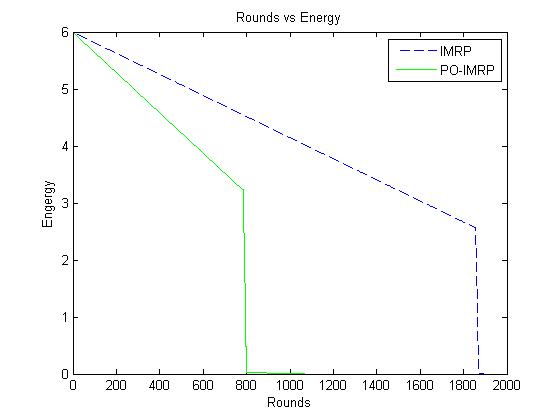

Supplement: Supplemental Information 1 [file peerj-cs-10-1908-s001.zip › IoT-OGI-code/OGI-code/figure/rounds vs energy.jpg]

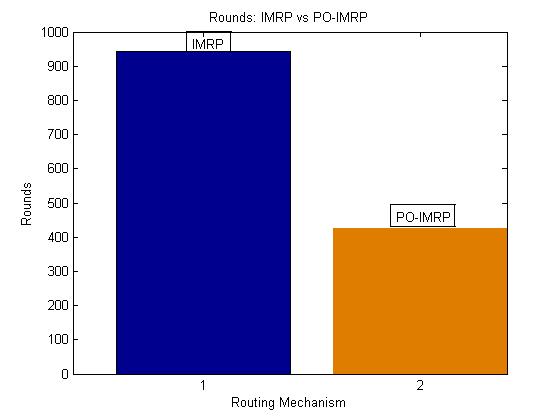

Supplement: Supplemental Information 1 [file peerj-cs-10-1908-s001.zip › IoT-OGI-code/OGI-code/figure/rounds vs mech.jpg]

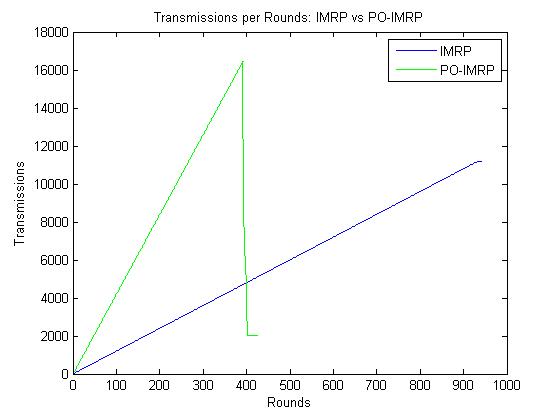

Supplement: Supplemental Information 1 [file peerj-cs-10-1908-s001.zip › IoT-OGI-code/OGI-code/figure/rounds vs packets.jpg]
